# Supplementary material for: Herpes zoster vaccine safety in the Aotearoa New Zealand population: a self-controlled case series study
Source: Nat Commun. 2023 Jul 19;14:4330. doi: 10.1038/s41467-023-39595-y (PMC10356758; doi:10.1038/s41467-023-39595-y)
Supplement: Supplementary file 1 — Supplementary Information [file 41467_2023_39595_MOESM1_ESM.pdf]

Supplementary information

Outcome Definitions

An Adverse event following immunisation: Any untoward medical occurrence that follows immunisation and does not necessarily have a causal relationship with the usage of the vaccine.[1]

Serious adverse events: An event that results in death is life-threatening, requires inpatient hospitalisation or prolongation of existing hospitalisation, results in persistent or significant disability/ incapacity, or is a congenital anomaly/birth defect.[1]

Injection site adverse events: Local reactions such as pain, redness, swelling, induration, pruritus, etc., at the injection site. [2]

Systematic adverse events: Generalised reactions such as headache, myalgia, fever, fatigue, etc. [2]

Cause-specific categorisation of adverse events following immunisation (Council for International Organisations of Medical Sciences and World Health Organisation)

Vaccine product-related reaction: An adverse event following immunisation that is caused or precipitated by a vaccine due to one or more of the inherent properties of the vaccine product.

Vaccine quality defect-related reaction: An adverse event following immunisation caused or precipitated by a vaccine that is due to one or more quality defects of the vaccine product, including its administration device as provided by the manufacturer.

Immunisation error related reaction: An adverse event following immunisation caused by inappropriate vaccine handling, prescribing or administration and thus, by its nature, is preventable.

Immunisation anxiety-related reaction: An adverse event following immunisation arising from anxiety about the immunisation.

Coincidental event: An adverse event following immunisation caused by something other than the vaccine product, immunisation error or immunisation anxiety, but a temporal association with immunisation exists and anxiety.

Table S1: Breakdown of tables that will be relevant to this study

	Datasets	Description
Demographic information	National Health Index (NHI)	Contains information needed to identify healthcare users, such as name, address (including domicile code), date of birth, sex, and ethnicity<br>Provides a mechanism for uniquely identifying every healthcare user by assigning each a unique number known as the NHI number
Zoster vaccination	National Immunisation Register (NIR)	Contains data derived from unit record immunisation event information. The NIR collection provides data for monitoring immunisation coverage and the progress of immunisation campaigns such as zoster.
Zoster and pre-specified adverse events	National Minimum Dataset (NMDS)	The NMDS is a national collection of public and private hospital discharge information, including clinical information, for inpatients and day patients. Unit record data is collected and stored. All records have a valid NHI number.
	Pharmaceutical Collection	It is a data warehouse that supports the management of pharmaceutical subsidies. It contains claim and payment information from pharmacists for subsidised dispensing that have been processed by the Sector Operations General Transaction Processing System.
	Mortality data	Data classifying the underlying cause of death for all deaths registered in New Zealand, including all registered foetal deaths (stillbirths), using the World Health Organization Rules and Guidelines for Mortality Coding. Information is broken down by age, sex, ethnicity, and district health board.
Covariates<br>- Comorbidities	Chronic condition/significant health event cohort	The dataset contains information about the healthcare users in the population cohort who meet the indicator requirements for one or more of eight chronic conditions.
Covariates<br>- Malignancies	Cancer registrations	Contains information on malignant cancer registrations, which can be used for cancer incidence and survival studies, public health research, monitoring screening programmes and policy formulation.
Covariates<br>- Income level	NZ deprivation index	Socioeconomic deprivation data and statistics [3, 4]. It is designed to measure relative socioeconomic deprivation, not absolute socioeconomic deprivation.<br>Purposes: resource allocation, research, and advocacy<br>It combines data related to<br>(a) Communication<br>(b) Income<br>(c) Employment<br>(d) Qualifications<br>(e) Owned home<br>(f) Support<br>(g) Living space<br>(h) Living condition
Ethnicity	Level 1 ethnic codes	1 European<br>2 Māori<br>3 Pacific Peoples<br>4 Asian<br>5 Middle Eastern/Latin American/African<br>6 Other Ethnicity<br>9 Residual Categories

Table S2: Immune-compromising conditions

Immune-compromising Condition	ICD-10 Code
Congenital (primary) immunodeficiency	
Cellular immune deficiencies: *T cell, natural killer T cell, mixed cellular and antibody defects, severe combined immune deficiency (SCID)*	D80: Immunodeficiency with predominantly antibody defects<br>D81: Combined Immunodeficiencies<br>D82: Immunodeficiency associated with other major defects.<br>D83: Common variable immunodeficiency<br>D84: Other immunodeficiencies
Acquired (secondary) immunodeficiency	
Malignant Hematologic Disorders: *Blood dyscrasia, leukaemia, lymphoma, other malignant neoplasms affecting the bone marrow or lymphatic systems*	C81: Hodgkin lymphoma<br>C82: Follicular lymphoma<br>C83: Non-follicular lymphoma<br>C84: Mature T/NK-cell lymphomas<br>C85: Other and unspecified types of non-Hodgkin lymphoma<br>C86: Other specified types of T/NK-cell lymphomas<br>C90: Multiple myeloma and malignant plasma cell neoplasms<br>C91: Lymphoid leukaemia<br>C92: Myeloid leukaemia<br>C93: Monocytic leukaemia<br>C94: Other leukaemias of specified cell type<br>C95: Leukemia of unspecified cell type<br>C96: Other and unspecified malignant neoplasms of lymphoid, hematopoietic and related tissue<br>D75.9: Disease of blood and blood-forming organs, unspecified
Human Immunodeficiency Virus (HIV) Infection	B20: HIV disease resulting in infectious and parasitic disease<br>B21: HIV disease resulting in malignant neoplasms<br>B22: HIV disease resulting in other specific diseases<br>B23: HIV disease resulting in other conditions<br>B24: Unspecified HIV disease
Post-Solid Organ Transplantation	Z94: Transplanted organ and tissue status
Post-Hematopoietic Stem Cell Transplantation	Z94.8: Other transplanted organ and tissue status
**Immunosuppressive drugs** (Name, Formulation ID)<br>Abiraterone acetate (404825)<br>Adalimumab (383625; 383626; 383627; 383628)<br>Aflibercept (409825)<br>Alectinib (412125)<br>Aminoglutethimide (105501)<br>Amsacrine (389725; 389726)<br>Anagrelide hydrochloride (384325)<br>Anastrozole (115801)<br>Anastrozole-DP (388625)	

Antithymocyte globulin (equine) (384125)
Antithymocyte Gobulin (375125; 375126)
Arsenic trioxide (384425; 384426; 384427)
Azacitidine (403425; 403426)
Azathioprine (110001; 110002; 110003; 110025)
Bacillus Calmette-Guerin (BCG) vaccine (392225; 392226)
Bendamustine hydrochloride (408425; 408426; 408427)
Bicalutamide (388325)
Bleomycin sulphate (381225; 381226)
Bortezomib (393625; 393626; 393627)
Busulfan (117301)
Calcium folinate (119801; 119802; 119803; 119804; 119825; 119826; 119827; 119828; 119829; 119830; 119831; 119832
Capecitabine (380825; 380826)
Carboplatin (382525; 382526; 382527; 382528; 382529)
Carmustine (383725; 383726)
Cetuximab (409425; 409426; 409427)
Chlorambucil (125501; 125502)
Ciclosporin (242101; 242102; 242103; 242125)
Cisplatin (382625; 382626; 382627)
Cladribine (384025; 384026; 384027
Colaspase [L-asparaginase] (383325; 383326)
Cyclophosphamide (136901; 136902; 136903; 136904; 136925; 136926)
Cytarabine (137101; 137102; 137103; 137104; 137125; 137126; 137127)
Dacarbazine (382725; 382726)
Dactinomycin [Actinomycin D] (384625; 384626)
Dasatinib (389425; 389426; 389427; 389428)
Daunorubicin (382825; 382826; 382827)
Docetaxel (383425; 383426; 383427; 383428; 383429; 383430; 383431)
Doxorubicin hydrochloride (381325; 381326; 381327; 381328; 381329; 381330)
Epirubicin hydrochloride (381425; 381426; 381427; 381428; 381429)
Erlotinib (391625; 391626)
Etanercept (378625; 378626; 378627)
Etoposide (243301; 243302; 243303; 243325)
Etoposide phosphate (384725; 384726)
Everolimus (404025; 404026)
Exemestane (387225)
Fludarabine phosphate (381025; 381026; 381027)
Fluorouracil (152902, 152903, 152904, 152925, 152926, 152927, 152928, 152929; 152930)
Flutamide (153701)
Fulvestrant (412625)
Gefitinib (396625)
Gemcitabine hydrochloride (384225; 384226; 384227; 384228)
Hydroxyurea [hydroxycarbamide] (162601)
Idarubicin hydrochloride (384825; 384826; 384827; 384828; 384829)
Ifosfamide (381925; 381926; 381927)
Imatinib mesylate (378025; 378026; 378027)
Infliximab (411425; 411426)
Interferon Alpha-2B (244505)
Interferon Gamma-1b (101801)

Irinotecan hydrochloride (381125; 381126; 381127)
Lapatinib ditosylate (394425)
Lenalidomide (403525; 403526; 403527; 403528)
Letrozole (118101)
Lomustine (383825; 383826)
Megestrol acetate (177201)
Melphalan (177301; 177302; 177325)
Mepolizumab (412725)
Mercaptopurine (178101; 178102; 178125)
Mesna (382025; 382026; 382027; 382028; 382029)
Methotrexate (179701; 179702; 179704; 179705; 179707; 179709; 179710; 179711; 179712; 179725; 179726; 179727; 179728; 179729; 179730; 179731; 179732; 179733; 179734)
Mitomycin C (384925; 384926; 384927; 384928; 384929)
Mitozantrone (382125; 382126; 382127; 382128)
Mycophenolate mofetil (103401; 103425; 103426; 103427)
Nilotinib (403825; 403826)
Nivolumab (406925; 406926; 406927)
Obinutuzumab (407825; 407826)
Octreotide (251101; 251102; 251103; 251104; 251105; 251106; 251126; 251127; 251128; 251129; 251130)
Octreotide (somatostatin analogue) (251201; 251202; 251203; 251204; 251205; 251206)
Octreotide LAR (somatostatin analogue) (251125; 251225; 251226; 251227)
Olaparib (251101; 251102; 251103; 251104; 251105; 251106; 251126; 251127; 251128; 251129; 251130)
Omalizumab (403925; 403926)
Oxaliplatin (383225; 383226; 383227; 383228; 383229)
Paclitaxel (381525; 381526; 381527; 381528; 381529; 381530)
Palbociclib (412525; 412526; 412527)
Pazopanib (394325; 394326)
Pegaspargase (400525; 400526)
Pembrolizumab (407225; 407226; 407227)
Pemetrexed (408825; 408826; 408827)
Pentostatin [Deoxycoformycin] (385025)
Pertuzumab (407725; 407726; 407727)
Procarbazine hydrochloride (204701)
Rituximab [Mabthera] (381725; 381726; 381727)
Rituximab [Riximyo] (412425; 412426; 412427)
Ruxolitinib (410625; 410626; 410627)
Secukinumab (410725)
Siltuximab (406425; 406426)
Sirolimus (376925; 376926; 386925; 386926; 386927)
Stilboestrol (218301)
Sunitinib (392125; 392126; 392127)
Tacrolimus (108801; 108802; 108825; 108826)
Tamoxifen citrate (221801; 221802)
Temozolomide (373325; 373326; 373327; 385625; 385626; 385627; 385628; 385629; 385630)
Teniposide (385125; 385126)
Thalidomide (384525; 384526)
Thioguanine (225201)

Thiotepa (225701; 225725)<br>Tocilizumab (411525; 411526; 411527; 411528)<br>Toremifene (371525)<br>Trastuzumab (381825; 381826; 381827)<br>Trastuzumab emtansine (412225; 412226; 412227)<br>Tretinoin (228625)<br>Venetoclax (412025; 412026; 412027; 412028)<br>Vinblastine sulphate (231901; 231925)<br>Vincristine sulphate (232001; 232002; 232025)<br>Vinorelbine (381625; 381626; 381627)

Table S3: Self-controlled case series (SCCS)

Study design	Effect measures	Information used in the analysis	Statistical analysis	Main assumptions
SCCS	Rate ratio	Every exposure and every event for each case patient	Poisson regression analysis	Events do not alter the probability of subsequent exposure, and events

Table S4: Classification and risk interval of pre-specified adverse events

Group	ICD-10-AM-iii Code	Description
**Group 1. Stroke, Cerebrovascular diseases**<br>(First episode in 12 months)<br>Risk window: 1–42 days		
Occlusion and stenosis	I443; I434	Occlusion and stenosis of precerebral arteries, Occlusion of cerebral arteries
Transient cerebral ischaemia	I435; G458-G468	Transient cerebral ischaemia; Other transient cerebral ischaemic attacks and related syndromes; Transient cerebral ischaemic attack, unspecified
Acute, and ill-defined, cerebrovascular disease	I436; I437	Acute, ill-defined cerebrovascular disease; Other and ill-defined cerebrovascular disease
Haemorrhage or Infarction	I60-I67	Subarachnoid haemorrhage; Intracerebral haemorrhage; Other nontraumatic intracranial haemorrhage; Cerebral infarction; Stroke, not specified as haemorrhage or Infarction
**Group 2. Cardiovascular events**<br>(First episode in 12 months)<br>Risk window: 1–42 days		
Acute myocardial infarction	I400; I411; I418; I210-I229	Acute myocardial infarction; Other acute and subacute forms of ischaemic heart disease
Acute pericarditis	I420; I308; I309	Acute pericarditis
Acute myocarditis	I422.0; I409; I401; I408; I514; I401-I422	Acute myocarditis in diseases classified elsewhere; Acute myocarditis NOS; Idiopathic myocarditis; Other and unspecified acute myocarditis; Myocarditis unspecified
Cardiomyopathy	I428; I430; I429; I422<br>I516-I519; I255-1256; I428	Other primary cardio-myopathies NOS; Cardiomyopathy in other diseases classified elsewhere; Secondary cardiomyopathy unspecified; Cardiomegaly-dilation, hypertrophy, ventricular dilation
Heart failure	I500; I501; I509	Congestive heart failure unspecified; Left heart failure; Heart failure unspecified
**Group 3. Meningitis, encephalitis, and encephalopathy**<br>(First episode in 12 months)<br>Risk window: 1–42 days		
Meningitis, encephalitis, and encephalopathy	A870; A89; G020; G028; G030; G050; G039; G04-G05; G934; G312; G92	Other unspecified viral meningitis; Unspecified viral infection of central nervous system<br>Meningitis in viral diseases classified elsewhere; Meningitis in other specified infectious and parasitic diseases classified elsewhere; Nonpyogenic meningitis

		Encephalitis, myelitis, and encephalomyelitis in other infectious and parasitic diseases classified elsewhere; Meningitis, unspecified; Encephalitis, myelitis, and encephalomyelitis. Encephalopathy, acute
**Group 4. Ramsay-Hunt Syndrome, Bell's Palsy**<br>(First episode in 12 months)<br>Risk window: 1–14 days		
Ramsay-Hunt Syndrome, Bell's Palsy	B022; G510; H490-H494, H510; G511, G519;	Geniculate herpes zoster,<br>Herpetic geniculate ganglionitis<br>Bell's palsy, Facial palsy
**Group 5. Medically attended Reactions**<br>(First episode in 30 days)<br>Risk window: 1–7 days		
Cellulitis and infection	L0310; L038; L039; M7969; M7989; T802; T888; T889; I889; I891; L049; R599	Cellulitis, upper arm, and forearm; Cellulitis<br>Cellulitis, unspecified site; Pain in limb; Limb swelling; Infection following infusion, infection, or vaccination; Complication of medical care; Nonspecific lymphadenitis, unspecified; Lymphadenitis; Acute lymphadenitis, unspecified; Enlarged lymph nodes, unspecified
Allergic reactions	T783; T887; T789; T784<br>L500; L501; T805<br>L501; T749; T782; T805	Angioneurotic oedema; Adverse effects of drugs; Adverse effect, unspecified; Allergy unspecified; Allergic urticaria; Idiopathic urticaria; Urticaria, unspecified; Serum reaction<br>Anaphylaxis

Type of encounter: Inpatient
Adapted from Safety of zoster vaccine in adults from a large managed-care cohort: a Vaccine Safety Datalink study [5].

Table S5: Falsification outcomes

Outcomes	ICD-10-AM-iii Code
Appendicitis	K350; K351; K359; K36; K37; K380; K381; K382; K383; K388; K389
Hernia	K4000; K4001; K4010; K4011; K4020; K4021; K4030; K4031; K4040; K4041; K4090; K4091; K410; K411; K412; K413; K414; K419; K420; K421; K429; K430; K431; K439; K440; K441; K449; K450; K451; K458; K460; K461; K469
Diverticulitis	K5700; K5701; K5702; K5703; K5710; K5711; K5712; K513; K5720; K5721; K5722; K5723; K5730; K5731; K5732; K5733; K5740; K5741; K5742; K5743; K5780; K5781, K5782, K5783; K5790; K57921; K5792; K5793;
Femoral fracture	S7200; S7201; S7202; S7203; S7204; S7205; S7208; S7210; S7211; S722; S723; S7240; S7241; S7242; S7243; S7244; S727; S728; S729
Cholelithiasis and cholecystitis	K8000; K8001; K8010; K8011; K8020; K8021; K8030; K8031; K8040; K8041; K8050; K8051; K8080; K8081; K810; K811; K818; K819; K820; K821; K822; K823; K824; K828; K829; K830; K831; K832; K833; K834; K835; K835; K838; K839; K870
Pancreatic diseases	K85; K860; K861; K862; K863; K868; K869; K871; K903
pertussis	A370; A371; A378; A379
Sepsis	A40-A41
Haemorrhoids	I840-I849; O224; O872
Renal calculi	N20-N23
Burns	M6137-M6139; T200-T3199

Table S6: Baseline characteristics of the study population before applying the outcome exclusion criteria.

Characteristics	Total<br>N = 1197	At risk<br>N = 370 (30.91%)	Control<br>N = 827 (69.09%)
**Years**			
2018	737 (61.57%)	240 (64.86%)	497 (60.10%)
2019	257 (21.47%)	78 (21.08%)	179 (21.64%)
2020	129 (11.37%)	35 (9.46%)	94 (10.78%)
2021	74 (6.89%)	17 (4.59%)	57 (6.18%)
**Age group**			
50 – 64	≤ 5 (0.33%)	≤ 5 (0.54%)	≤ 5 (0.24%)
65 – 69	288 (24.06%)	89 (24.05%)	199 (24.06%)
70 – 74	356 (29.74%)	117 (31.62%)	239 (28.90%)
75 - 76	425 (35.51%)	132 (35.68%)	293 (35.43%)
≥ 80	124 (10.36%)	30 (8.11%)	94 (11.37%)
**Sex**			
Male	706 (58.98%)	211(57.03%)	495 (59.85%)
Female	491(41.02%)	159 (42.97%)	332 (40.15%)
**Ethnicity (Level 1 ethnic codes)**			
European (1)	861(71.93%)	254 (68.65%)	607 (73.40%)
Māori (2)	167 (13.95%)	62 (16.76%)	105 (12.70%)
Pacific Peoples (3)	70 (5.85%)	21 (5.68%)	49 (5.93%)
Asian (4)	84 (7.02%)	27 (7.30%)	57 (6.89%)
Others (5,6,9)	15 (1.25%)	6 (1.62%)	9 (1.09%)
**NZDep2013** (Quintiles)			
Quintile 1	182 (15.20%)	52 (14.05%)	130 (15.72%)

Quintile 2	186 (15.54%)	50 (13.51%)	136 (16.45%)
Quintile 3	237 (19.80%)	81 (21.89%)	156 (18.86%)
Quintile 4	274 (22.89%)	86 (23.24%)	188 (22.73%)
Quintile 5	318 (26.57%)	101 (27.30%)	217 (26.24%)
**NZDep2018** (Quintiles)			
Quintile 1	172 (14.37%)	49 (13.24%)	123 (14.87%)
Quintile 2	208 (17.38%)	59 (15.95%)	149 (18.02%)
Quintile 3	248 (20.72%)	80 (21.62%)	168 (20.31%)
Quintile 4	269 (22.47%)	84 (22.70%)	185 (22.37%)
Quintile 5	300 (25.06%)	98 (26.49%)	202 (24.43%)
**Immune suppression**			
Yes	101 (8.44%)	33 (8.92%)	68 (8.22%)
No	1096 (91.56%)	337 (91.08%)	759 (91.78%)
**District Health Board**			
Auckland	90 (7.52%)	30 (8.11%)	60 (7.26%)
Bay of Plenty	62 (5.18%)	17 (4.59%)	45 (5.44%)
Canterbury	143 (11.95%)	47 (12.70%)	96 (11.61%)
Capital & Coast	54 (4.51%)	16 (4.32%)	38 (4.59%)
Counties Manukau	128 (11.69%)	46 (12.43%)	82 (9.92%)
Hawke's Bay	38 (3.17%)	10 (2.70%)	28 (3.39%)
Hutt Valley	46 (3.84%)	14 (3.78%)	32 (3.87%)
Lakes	32 (2.67%)	8 (2.16%)	24 (2.90%)
MidCentral	63 (5.26%)	22 (5.95%)	41 (5.00%)
Nelson Marlborough	32 (2.67%)	11 (2.97%)	21 (2.54%)
Northland	58 (4.85%)	17 (4.59%)	41(4.96%)
South Canterbury	17 (1.42%)	≤ 5 (0.81%)	14 (1.69%)
Southern	92 (7.69%)	25 (6.76%)	67 (8.10%)
Tairawhiti	10 (0.84%)	≤ 5 (0.27%)	9 (1.09%)
Taranaki	17 (1.42%)	≤ 5 (1.35%)	12 (1.45%)
Waikato	125 (10.44%)	30 (8.11%)	95 (11.49%)

Wairarapa	12 (1.00%)	≤ 5 (0.54%)	10 (1.21%)
Waitemata	132 (11.03%)	50 (13.51%)	82 (9.92%)
West Coast	18 (1.50%)	9 (2.43%)	9 (1.09%)
Whanganui	28 (2.34%)	7 (1.89%)	21(2.54%)

New Zealand Index of Deprivation (2013 and 2018) measures relative socioeconomic deprivation. It combines data related to communication, income, employment, qualifications, house ownership, support, living space and living condition. It is divided into quintiles (Quintile 1= least deprived; Quintile 5=most deprived), with each quintile representing 20% of the population.
At risk: Vaccine recipients with adverse events within 1 to 42 days post-vaccination.
Control: Vaccine recipients with adverse events within 73 to 162 days post-vaccination.

BIBLIOGRAPHY

1. World Health Organization. Global manual on surveillance of adverse events following immunisation. 2016.
2. Tricco AC, Zarin W, Cardoso R, Veroniki A-A, Khan PA, Nincic V, et al. Efficacy, effectiveness, and safety of herpes zoster vaccines in adults aged 50 and older: systematic review and network meta-analysis. BMJ. 2018;363:k4029.
3. Atkinson J, Salmond C, Crampton P. NZDep2013 index of deprivation. Wellington: Department of Public Health, University of Otago. 2014.
4. Atkinson J, Crampton P, Salmond C. NZDep2018 Index of Deprivation, Interim Research Report, December 2019. Wellington: University of Otago; 2019.
5. Tseng HF, Liu A, Sy L, Marcy SM, Fireman B, Weintraub E, et al. Safety of zoster vaccine in adults from a large managed-care cohort: a Vaccine Safety Datalink study. Journal of Internal Medicine. 2012;271(5):510-20.
