# Supplementary material for: Herpes zoster vaccine safety in the Aotearoa New Zealand population: a self-controlled case series study
Source: Nat Commun. 2023 Jul 19;14:4330. doi: 10.1038/s41467-023-39595-y (PMC10356758; doi:10.1038/s41467-023-39595-y)
Supplement: Supplementary file 3 — Reporting Summary [file 41467_2023_39595_MOESM3_ESM.pdf]

nature portfolio

Corresponding author(s): Dr James F. Mbinta and Prof Colin R. Simpson

Last updated by author(s): Jun 12, 2023

Reporting Summary

Nature Portfolio wishes to improve the reproducibility of the work that we publish. This form provides structure for consistency and transparency in reporting. For further information on Nature Portfolio policies, see our Editorial Policies and the Editorial Policy Checklist.

Statistics

For all statistical analyses, confirm that the following items are present in the figure legend, table legend, main text, or Methods section.

n/a	Confirmed	
☐	☒	The exact sample size (*n*) for each experimental group/condition, given as a discrete number and unit of measurement
☐	☒	A statement on whether measurements were taken from distinct samples or whether the same sample was measured repeatedly
☒	☐	The statistical test(s) used AND whether they are one- or two-sided<br>*Only common tests should be described solely by name; describe more complex techniques in the Methods section.*
☐	☒	A description of all covariates tested
☐	☒	A description of any assumptions or corrections, such as tests of normality and adjustment for multiple comparisons
☒	☐	A full description of the statistical parameters including central tendency (e.g. means) or other basic estimates (e.g. regression coefficient) AND variation (e.g. standard deviation) or associated estimates of uncertainty (e.g. confidence intervals)
☒	☐	For null hypothesis testing, the test statistic (e.g. *F*, *t*, *r*) with confidence intervals, effect sizes, degrees of freedom and *P* value noted<br>*Give P values as exact values whenever suitable.*
☒	☐	For Bayesian analysis, information on the choice of priors and Markov chain Monte Carlo settings
☒	☐	For hierarchical and complex designs, identification of the appropriate level for tests and full reporting of outcomes
☒	☐	Estimates of effect sizes (e.g. Cohen's *d*, Pearson's *r*), indicating how they were calculated

Our web collection on statistics for biologists contains articles on many of the points above.

Software and code

Policy information about availability of computer code

Data collection	We used routinely collected data available at the Ministry of Health
Data analysis	All code used in this study is publicly available at Vaccine-studies (doi:10.5281/zenodo.8022695). The analyses were carried out by JM and AW and independently checked by BN and SB. All statistical analyses were carried out using R/R Studio (version R-4.0.5) and python (version 3.7.12).

For manuscripts utilizing custom algorithms or software that are central to the research but not yet described in published literature, software must be made available to editors and reviewers. We strongly encourage code deposition in a community repository (e.g. GitHub). See the Nature Portfolio guidelines for submitting code & software for further information.

Data

Policy information about availability of data

All manuscripts must include a data availability statement. This statement should provide the following information, where applicable:

- Accession codes, unique identifiers, or web links for publicly available datasets
- A description of any restrictions on data availability
- For clinical datasets or third party data, please ensure that the statement adheres to our policy

We used routinely collected national data available at the Ministry of Health. The National Health Index was used to link data extracted from multiple datasets. Clinical, discharge and demographic information of hospitalised patients (in-patients and day patients in public and private hospitals) were obtained from the national minimum datasets. Detailed clinical and pathological data (morphology code, basis of diagnosis, laboratory code, and extent of disease) about cancer and demographic information of patients with malignancy were extracted from the New Zealand cancer registrations. The national immunisation register (NIR) is an accurate electronic medical record that contains immunisation information on childhood and adult vaccines administered in New Zealand.
The data used in this study are based on de-identified national clinical records and will not be made publicly available. These are, however, available by application via the Ministry of Health New Zealand.

Human research participants

Policy information about studies involving human research participants and Sex and Gender in Research.

Reporting on sex and gender	The present study included sex (males and females) as reported by the Ministry of Health.
Population characteristics	These baseline characteristics included<br>[1] Age: All adults involved in the study were 50 years and older. Age was divided into five age bands (50–64; 65–69; 70–74; 75-76; ≥ 80).<br>[2] Sex: This was divided into males and females as reported by the Ministry of Health.<br>[3] Ethnicity: This was based on best practice for recording ethnicity within health provider information systems and the National Health Index, and reporting ethnicity to National Collections. We used level 1 ethnic codes (1 = European; 2 = Maori; 3 = Pacific Peoples; 4 = Asian; 5 = Middle Eastern/Latin American/African; 6 = Other Ethnicity; 9 = Residual Categories). 5, 6, and 9 were combined because of small numbers.<br>[4] Index of deprivation: This is the socioeconomic deprivation data and statistics. It is designed to measure relative socioeconomic deprivation, not absolute socioeconomic deprivation. It is an ordinal scale and ranges from 1 to 10, where 1 represents the areas with the least deprived scores and 10 the areas with the most deprived scores. For this study, we divided the index of deprivation into quintiles (quintile 1 being the least deprived and quintile 5 being the most deprived)<br>[5] Location: This was based on District Health Boards. These boards are responsible for providing or funding the provision of health services in their district.<br>[6] Immunosuppression: This was treated as a binary variable (immunosuppressed =1, immunocompetent = 0) based on congenital (primary) immunodeficiency, and acquired (secondary) immunodeficiency.
Recruitment	In step one, we identified all individuals who had completed herpes zoster vaccination between 1st April 2018 and 24th July 2021 and were born in 1971 or earlier.<br>In step two, we identified all individuals who had a publicly funded hospital discharge where the date of admission fell within 42 days (inclusive) of the herpes zoster vaccination date (index date) and there was any diagnosis of any of the prespecified outcomes. We considered the date of admission as the day of onset of the adverse event. For each master NHI number identified in step two, we eliminated all publicly funded hospital discharges with an admission date that falls within one year of admission date of the index event (except for group five adverse events). This was to ensure that the adverse event identified was the first event in 12 months (except for group five, where we considered the first event in 30 days).<br>For each master encrypted NHI number with a completed herpes zoster vaccination identified in step two, we extracted all publicly funded hospital discharges with an admission date that falls within 73 and 162 days (inclusive) of the vaccination date. For each master NHI number, we excluded all publicly funded hospital discharges with an admission date that falls within one year of admission date of the index event (except group five adverse events). In scenarios where a person has more than one index event each index event had its year of retrospective data included.<br>The final study population comprised of individuals with the outcome of interest that occurred during the follow-up (predefined observation) period. We were interested in who and when hence controlling for time-fixed confounding variables. For each outcome of interest, we noted the event ID, start date, clinical code, and diagnosis type (primary or secondary diagnosis).
Ethics oversight	An ethics exemption (21/NTB/118) was obtained from the Health and Disability Ethics Committee of the Ministry of Health, New Zealand.

Note that full information on the approval of the study protocol must also be provided in the manuscript.

Field-specific reporting

Please select the one below that is the best fit for your research. If you are not sure, read the appropriate sections before making your selection.

☒ Life sciences ☐ Behavioural & social sciences ☐ Ecological, evolutionary & environmental sciences

For a reference copy of the document with all sections, see nature.com/documents/nr-reporting-summary-flat.pdf

Life sciences study design

All studies must disclose on these points even when the disclosure is negative.

Sample size	From April 2018 to July 2021, 278,375 adults were vaccinated with the zoster vaccine live and were included in the study. All the vaccinated people were followed up during the at risk and control periods. All vaccinated people who developed an outcome of interest during the

	follow-up period were included in the analysis. This was a nationwide study and therefore sample size calculation was not necessary.
Data exclusions	All adults without a record of herpes zoster vaccination between 1 April 2018 and 24th July 2021 were excluded from the study.
Replication	Replication was successful. The analyses were carried out by two people and independently checked by two statisticians. All code used in this study is publicly available at Vaccine-studies (doi:10.5281/zenodo.8022695).
Randomization	This was an observational study based on routinely collected data available at the Ministry of Health. Randomization is not applicable in self-controlled case series studies due to the design's focus on within-individual comparisons over time. We compared the occurrence of an serious adverse events during at-risk and control periods within the same individual, eliminating the need for random assignment to different groups.<br>In the analysis, we compared the occurrence of an event during at-risk and control periods within each individual. The at-risk period was identified based on changes in exposure status (after the administration of the vaccine.) The same individual serves as their own control, eliminating the need for randomization.
Blinding	Blinding was not relevant to our study (self controlled case series) for the following reasons:<br>[1] The comparison was made within the same individual over time, focusing on the occurrence of serious adverse events during the at-risk and control periods. Since each individual served as their own control, blinding was not applicable because the exposure status was inherently known to the research team.<br>[2] The exposure (vaccination) and serious adverse events were assessed through medical databases, where blinding was not feasible.

Reporting for specific materials, systems and methods

We require information from authors about some types of materials, experimental systems and methods used in many studies. Here, indicate whether each material, system or method listed is relevant to your study. If you are not sure if a list item applies to your research, read the appropriate section before selecting a response.

Materials & experimental systems

n/a	Involved in the study
☒	☐ Antibodies
☒	☐ Eukaryotic cell lines
☒	☐ Palaeontology and archaeology
☒	☐ Animals and other organisms
☒	☐ Clinical data
☒	☐ Dual use research of concern

Methods

n/a	Involved in the study
☒	☐ ChIP-seq
☒	☐ Flow cytometry
☒	☐ MRI-based neuroimaging
